# Activation of RIG-I signaling to increase the pro-inflammatory phenotype of a tumor

**DOI:** 10.18632/oncotarget.26729

**Published:** 2019-03-22

**Authors:** David L. Elion, Rebecca S. Cook

**Affiliations:** Cancer Biology Graduate Program, Vanderbilt University School of Medicine, Nashville, TN, USA; Department of Cell and Developmental Biology, Vanderbilt University School of Medicine, Nashville, TN, USA; Department of Biomedical Engineering, Vanderbilt University School of Engineering, Nashville, TN, USA; Vanderbilt Ingram Cancer Center, Vanderbilt University Medical Center, Nashville, TN, USA

**Keywords:** immunotherapy, RIG-I, intrinsic immunity, apoptosis, pyroptosis

Immune checkpoint inhibitors (ICIs) are achieving remarkable successes in several cancers, including melanoma, colon, and lung cancers. However, these same successes for ICIs are not yet being realized in estrogen receptor positive (ER+) breast cancer, the most common breast cancer subtype. Compared to triple-negative breast cancer (TNBC), ER+ breast cancers exhibit lower overall response rates to ICI treatments, including anti-programmed cell death (PD)-1, and anti-PD-ligand 1 (PD-L1) [1]. Accumulating evidence suggests that TNBCs harbor more tumor infiltrating lymphocytes (TILs), a higher mutational burden, and higher tumor cell PD-L1 expression as compared to ER+ breast cancers, characteristics that may prime TNBCs for ICI response [2]. If so, then it is possible that treatment strategies aimed at increasing TILs in the tumor microenvironment (TME) of ER+ breast cancers might increase ICI responsiveness [3]. This hypothesis underlies intense research efforts to identify treatment strategies that would increase TILs in ER+ breast cancers, potentially enabling effective and durable ICI responses.

Numerous reports demonstrate that most cells in the body, including tumor cells as well as cells of the TME, express receptors that recognize and respond to viral nucleotide motifs, referred to as pattern recognition receptors (PRRs) [4]. PRRs are an important element of innate immunity. Once activated, PRRs initiate signaling pathways that generate a pro-inflammatory microenvironment that becomes replete with lymphocytes. Therapeutic approaches that use non-infectious methods to activate PRR signaling within the TME is gaining momentum as a strategy for priming tumors for ICI sensitivity, with much excitement surrounding agonists of the PRR known as STING [4]. We recently investigated the PRR known as retinoic acid-inducible gene (RIG)-I in models of ER+ breast cancers, testing the possibility that, when appropriately delivered and modulated, RIG-I mimetics might have robust therapeutic as a cancer treatment, as is currently being explored for STING agonists. We found that RIG-I signaling resulted in potent TIL recruitment to the TME, and primed otherwise insensitive tumors for sensitivity to PD-L1 treatment [4, 5].

RIG-I is a cytosolic RNA helicase, recognizing RNA motifs specific to certain viruses. Years of RIG-I research has culminated in the discovery of synthetic non-infectious RIG-I mimetics, comprised of minimal stem-loop RNA (SLR) sequences harboring a 5′-triphosphate motif, capable of potent RIG-I activation in cultured tumor cells and in mice *in vivo* [6]. Delivery of SLR sequences to cultured ER+ breast cancer cells activated RIG-I signaling, and induced immunogenic programs of breast cancer cell death, including pyroptosis and extrinsic apoptosis [5], demonstrating that RIG-I signaling has important therapeutic consequences that occur in a breast cancer cell intrinsic manner. Interestingly, we also found that RIG-I activation using SLR sequences induced expression of pro-inflammatory and T-cell recruiting cytokines by ER+ breast cancer cells. Further, RIG-I signaling in response to SLR sequences increased expression of Major Histocompatibility (MHC)-I proteins by ER+ breast cancer cells, and increased breast cancer cell expression of PD-L1. Together, these observations support the hypothesis that therapeutic RIG-I activation might recruit TILs and prime the TME for ICI response.

The effective delivery of SLR sequences to the TME *in vivo* was enabled by recent advances in nanoparticle (NP)-mediated delivery of RNA interference (RNAi) technologies. We focused on a previously described NP design strategy used with siRNAs, based on its proven *in vivo* protection of siRNA sequences, longer circulating half-life, increased uptake of siRNA by tumor cells, and enhanced endosomal release of siRNA cargo into the cytoplasm of tumor cells [7]. Adapting this NP design for use with SLR sequences, we found that NP-SLR delivery activated RIG-I signaling in tumor cells *in vivo* [5]. Similar to what was seen in cultured breast cancer cells, we found that RIG-I activation in tumor cells *in vivo* resulted in increased expression of pro-inflammatory cytokines. Importantly, we found increased CD4+ and CD8+ TILs and heightened ICI sensitivity in tumors treated with NP-SLR, consistent with the larger hypothesis that activation of innate immunity in ER+ breast cancers may prime these tumors for response to ICI.

These findings support clinical translation of RIG-I agonists, which is currently underway (NCT03065023) using the RIG-I agonist from Merck, MK-4621, in advanced and recurrent tumors. Another clinical trial recently opened (NCT03739138) to identify the therapeutic effects of MK-4621 with anti-PD-1 (Pembrolizumab) in patients with advanced and recurrent tumors. These trials are supported by our data demonstrating that RIG-I agonists increase ICI response in breast cancers through at least two mechanisms, tumor intrinsic apoptosis and enhanced immunogenicity of the TME. It is anticipated that, although RIG-I agonists are only in earliest phases of exploration as cancer treatment strategy, the field will move forward at a rapid pace, based on the vast potential for its success in immune-oncology.
